# In the beginning when the Japanese Environmental Mutagen Society was established

**DOI:** 10.1186/s41021-016-0052-x

**Published:** 2016-12-01

**Authors:** Takashi Sugimura

**Affiliations:** 1 The Japan Academy, 7-32, Ueno Park, Taito-ku, Tokyo Japan; 2 National Cancer Center Research Institute, 1-1, 5-chome, Chuo-ku, Tokyo Japan

## Abstract

Upon the commemoration of the 10th anniversary of the journal, “Genes and Environment”, the history of sciences on environmental mutagens in Japan was reviewed.

The history of sciences on environmental mutagens was initiated from the discovery of a high incidence of scrotum cancers in chimney sweeps in London by Pott, P in 1775. Based on this, Yamagiwa, K, Tokyo, carried out repeated painting of coal tar on the ears of rabbits, and found the production of skin carcinomas in 1915, this being the first successful experiment on chemical carcinogenesis.

Following this, a British group represented by Kennaway, EL fractionated tar and purified an active principle, dibenz[*a.h*]anthracene, a polycyclic aromatic hydrocarbon (PAH), to produce cancer by painting it on the back skin of mice. This is the first evidence that pure substances from our environment produced cancers. Meanwhile, Gelboin H at NCI, NIH USA and Weinstein B at Columbia University found that benzo[*a*]pyrene (BP), a PAH, binds to tissue proteins and nucleic acids. In England too, a group represented by Brookes, P & Lawley, PD studied the binding of BP with tissue components. Miller, EC and Miller, JA, the McArdle Institute, Wisconsin found that a typical carcinogen 2-acetylaminofluorene (2AAF) binds to DNA and protein after activation by microsomal and soluble enzymes.

During these periods, it was revealed that carcinogens were metabolized by enzymes, including cytochrome P450, and converted to reactive intermediates. On the other hand, epidemiological studies revealed the close relation between environments and cancer incidences. A typical example was that immigrants to Hawaii from Japan showed a higher incidence of colon cancers. Involvement of occupational conditions with cancer incidence is also a typical example of environmental carcinogenesis.

With an understanding of the above series of evidence, Ames, B. California invented a useful method to detect mutagens and carcinogens in 1973. It is based on incubation of the histidine requirement mutant of bacteria (*Salmonella typhimurium*), fractions of microsomes and supernatants from the liver of rats and test substances. This rapid method to detect revertants of microbes within a couple of days was used all over the world. Ames held the first International Conference on Environmental Mutagens (ICEM) in Asilomar California in 1973. Many substances, including known carcinogens, were tested. Ames’s group, Sugimura’s group and other groups-analyzed vast numbers of environmental substances and demonstrated that most carcinogens are mutagens. These results made an impact on researchers on carcinogenesis studies in the world.

The second ICEM was held in Edinburgh, Scotland by Prof. Bridges, B in 1977, and the third one was held in Japan, Tokyo (Sugimura, T), Mishima (Kada, T) and Kyoto (Dr. Nishioka, H) in 1981. The fourth one was held in Stockholm by Prof. Ramel, C in 1985. Kada emphasized the importance of substances that inhibit mutagenesis. So-called anti-mutagens in vegetables were demonstrated and later found to be polyphenol compounds.

In Japan, the presence of 4NQO (4-nitroquinoline 1-oxide) synthesized by Ochiai, E, Tokyo, during the Second World War in experiments as anti-malaria drugs gave us a very convenient tool. 4NQO was metabolized to 4-hydroxyaminoquinoline 1-oxide (4-HAQO) by NAD[P]H dehydrogenase. No requirement of the cytochrome P450 system simplifies the genetical analyses of 4NQO commonly in bacteria and mammalian cell systems.

During the 1960s, AF2 (acryl furylfuramide) was widely used as a bactericidal food additive in Japan, based on a report of non-carcinogenicity. However, Kondo, S and Kada, T found that AF2 was a very strong mutagen in *E. coli* and *B. subtilis*, respectively. However, it was not mutagenic in the original series of *S. typhimurium* tester strains reported by Ames B. Then, largescale precise carcinogenesis experiments demonstrated the carcinogenicity of AF2. Usage of AF2 as a food additive was immediately banned. Meanwhile Ames modified his tester strains of *S. typhimurium* by introducing plasmids.

Although a typical carcinogen, *N*,*N*-dimethylnitrosamine was not mutagenic by the improved Ames method, and Yahagi, T and Matsushima, T further modified the method by pre-incubating bacteria, chemical and metabolizing enzymes. Since AF2 was widely used in Japan, the importance of the mutagenicity of environmental substances was well understood.

Soon after, we found the presence of many strong mutagens in grilled fish and meat, and many new compounds, such as heterocyclic amines (HCA) in the 1980s. They include imidazoquinoline (IQ), imidazoquinoxaline (IQx), phenyl-imidazopyridine (PhIP) and others. They were proven to be carcinogenic in rodents.

Meanwhile during the study of DNA adducts of HCAs, Kasai, H and Nishimura, S discovered 8-hydroxyguanine. It was an oxidative product in DNA and RNA.

Nowadays, as a cause of cancer, viral infections (Hepatitis B and C viruses, Papilloma viruses), and bacterial infection (*Helicobactor pylori*) can explain about 20 % of total human cases. Hormone involvement also is being considered as a candidate. However, cases of gastric cancer appear in only less than 5 % of stomachs infected with *Helicobactor pylori*. It has been clarified that 70 ~ 80 % of Japanese gastric cancers are associated with *H. pylori* infection. *H.pylori* itself might not be sufficient to develop gastric cancer, and many unidentified chemicals (environmental mutagens/carcinogens) might be involved. Formation of stomach cancer in Mongolian gerbils infected with *H. pylori* is enhanced very much by the administration of *N*-methyl-*N*’-nitro-*N*-nitrosoguanidine (MNNG).

Cancer is involved with many gene mutations in a single cell. The number of combinations of mutated genes is countless. Even among a single tumor, their combinations are heterogeneous. The environmental mutagens also consist of numerous substances in very limited concentrations. However, their presences cannot be overlooked, since their effects would accumulate for the final completion of carcinogenesis.

The Environmental Mutagen Society plays an extremely important role in the progress of studies of cancer causes and their related gene alterations, cancer prevention and aging. We hope that the Japanese Environmental Mutagen Society will further develop and contribute to the health and welfare of society.

